# Education Research: Simulator-Based Training in Transcranial Doppler Ultrasound: Enhancing Competency in a Critical Neuroimaging Tool

**DOI:** 10.1212/NE9.0000000000200285

**Published:** 2026-01-27

**Authors:** Aidan Shev, Florence Sheehan, Ziao Yin, Angela Straccia, Daniel F. Leotta, Alberto Aliseda, R. Eugene Zierler, Mark D. Anderson, Deepak Sharma

**Affiliations:** 1 Medicine, University of Washington, Seattle;; 2 Mechanical Engineering, University of Washington, Seattle;; 3 Applied Physics Laboratory, University of Washington, Seattle;; 4 Vascular Surgery, University of Washington, Seattle; and; 5 Anesthesiology and Pain Medicine, University of Washington, Seattle.

## Abstract

**Background and Objectives:**

Transcranial Doppler (TCD) ultrasound is recommended for measuring cerebral blood flow velocity to guide therapeutic intervention in patients with cerebrovascular diseases. However, the widespread use of TCD is hampered by a shortage of trained practitioners. This study evaluated the efficacy of a simulator-based curriculum in teaching novices the foundational skill to perform TCD insonation.

**Methods:**

We developed a curriculum on a novel simulator that incorporates interactive practice with real-time feedback. We assessed change in the skill levels of medical and premedical students without previous exposure to TCD from pretraining and posttraining tests. Psychomotor skill was assessed based on accuracy in insonating the middle cerebral, anterior cerebral, ophthalmic, basilar, and vertebral arteries. Cognitive skill (knowledge) was assessed using multiple-choice questions (MCQs).

**Results:**

Training significantly improved skill: the correct insonation rate increased from 16 ± 24% (95% CI 9%–23%) on the pretest to 61 ± 34% (95% CI 52%–70%) on the posttest (N = 56, *p* < 0.0001, Cohen d = 1.5). First-attempt accuracy improved from 15 ± 23% (95% CI 9%–21%) to 49 ± 31% (95% CI 41%–57%) (N = 56, *p* < 0.0001, Cohen d = 0.9). Knowledge increased markedly, with MCQs answered correctly rising from 7 ± 26% (95% CI 0%–14%) to 64 ± 19% (95% CI 59%–69%) (*p* < 0.0001, Cohen d = 2.5). The course, including pre-test and posttests, was completed in 2.6 ± 1.0 hours.

**Discussion:**

Simulator-based training rapidly improved the ability of novices to locate intracranial arteries for measurement of blood flow velocity. The data support the employment of simulator-based training as a foundational step to help alleviate the shortage of trained providers. The simulator's self-directed practice allows for scalable, standardized training.

## Introduction

Transcranial Doppler (TCD) provides real-time, continuous measurement of intracranial arterial blood flow velocities safely and noninvasively at the bedside.^1,2^ The TCD probe is placed over acoustic windows where ultrasound is able to penetrate the skull. Evidence-based clinical applications of TCD include the assessment of cerebral vasospasm, stroke, sickle cell disease (SCD), and brain death, among others.^3-5^ Despite these indications, TCD utilization remains significantly limited globally and within the United States, even with rapid adoption of ultrasound across diverse medical disciplines.^6-8^ The limitation largely stems from the scarcity of adequately trained practitioners, the complex nature of TCD, and the challenges associated with practicing on critically ill patients.^1,6-9^

We previously demonstrated the efficacy of a transthoracic echo simulator that we built for training residents, medical students, and nurse practitioners.^10,11^ In response to the urgent need for more skilled TCD operators, we built a TCD simulator and developed a curriculum that integrates foundational knowledge with practical, hands-on experience. This study was performed to validate our simulator-based curriculum's efficacy in teaching the foundational skills of nonimaging TCD, specifically vessel identification and insonation. In nonimaging TCD, insonation must be inferred indirectly from observation of power M-mode and spectral waveform displays. However, only nonimaging TCD allows continuous cerebral blood flow velocity display and emboli monitoring as needed in the perioperative period. Therefore, this simulator focused on training in nonimaging TCD.

## Methods

### Study Population

Medical and premedical students at the University of Washington (UW) were offered free training in TCD to help evaluate a new curriculum. Eligibility criteria were age ≥18 years and lack of prior experience in TCD. We determined that a sample size of 25 would detect a 33% success rate in insonation after training with 80% power, assuming a 0% baseline success rate. However, we aimed to recruit 30 students to allow for potential dropouts and ensure robustness.

### Simulator Design

**Figure 1:**
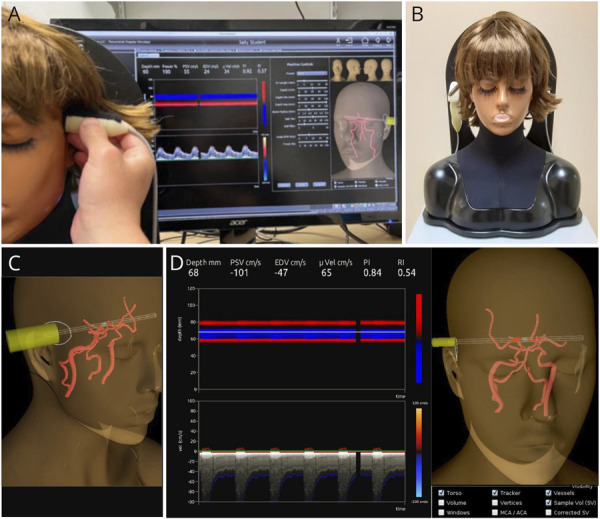
Transcranial Doppler Simulator With Visual Guidance (A) The mannequin (left) is being scanned with the mock transducer. On the left panel of the computer monitor, the power M-mode and spectral waveform displays morph with probe manipulation in real time. Machine controls are on the center panel. On the right panel is visual guidance, an interactive 3D tool that displays a map of the intracerebral arteries, probe, ultrasound beam's path, artery intersections, and the sample volume. (B) The mannequin can be scanned either upright or supine. (C) Details of visual guidance showing the probe, ultrasound beam entering the skull at the right temporal acoustic window (white circle), and sample volume (blue) within an insonated artery segment. (D) Details of visual guidance showing that the beam is insonating 3 artery segments (highlighted in red), resulting in 3 bars in the power M-mode display. Blood flow velocity is being measured at a depth of 68 mm from the probe and displayed on the spectral waveform. The measurement depth is set by the user. Note the box at the base of the 3D display: trainees can hide the mannequin, vessels, beam, probe, and sample volume using the Visibility controls to simulate scanning a patient.

**Figure 2:**
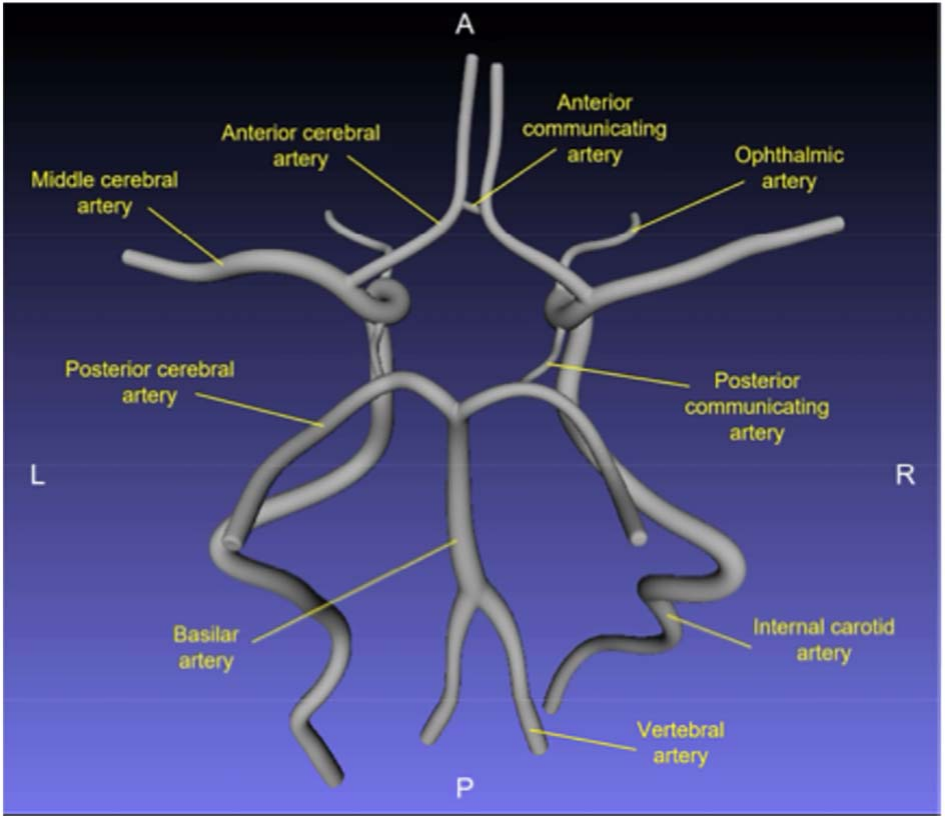
The Circle of Willis 3D surface model of the Circle of Willis prepared for computational fluid dynamics (CFD) modeling. Reproduced with permission.^12^

The simulator (TCD Simulator, Sheehan Medical LLC, Mercer Island WA) (Figure 1) consists of a mannequin, a mock transducer (probe), and a computer. Probe position is tracked using a magnetic field system (Patriot, Polhemus, Colchester VT). This high-fidelity system is more than a task trainer; it integrates patient-specific anatomy and hemodynamics with diagnostic tools, allowing for practice in both introductory procedural skills and clinical scenarios. To “scan” a patient, the student manipulates the probe on the mannequin to position a virtual pulsed-Doppler sample volume in an intracranial artery (Figure 1C). The Doppler measurements at the insonation point are indicated on the computer in real time through a power M-mode display of flow signal intensity and direction, a spectral waveform display of flow velocity, and audio (Figure 1D). The simulator's library contains 27 cases, each developed from a CT angiography (CTA) scan and a TCD examination of patients at the University of Tennessee (N = 2), Vanderbilt University (N = 1), or the UW (N = 24). The CTA was segmented to obtain a 3D reconstruction of the included arteries, which was then truncated to create a model of the intracerebral vessels.^12^ The arterial segments included in the 3D model are displayed in Figure 2. Computational fluid dynamics (CFD) modeling was then applied to the reconstruction to calculate 4D (3D spatially and temporally resolved) flow velocities within the vascular lumen, using TCD data for initialization.^13^ To establish clinical validity, an expert compared the Doppler spectral waveforms generated by the simulator with the patients' clinical TCD images, and the CFD model was modified as needed to address discrepancies.

### Simulator Training Using Visual Guidance

**Figure 3:**
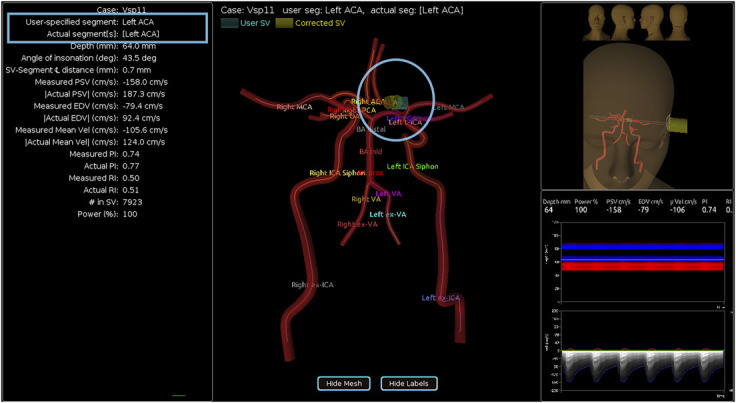
Vessel Segment Acquisition Feedback This acquisition feedback screen reports the student-specified segment and the actual segment acquired in text (upper left) and graphically on the 3D reconstruction of the patient's Circle of Willis (circle). The feedback helps guide the learner on their next acquisition.

Visual guidance (Figure 1) is an interactive 3D tool that shows the ultrasound beam's path through the Circle of Willis, its intersection(s) with artery segments (highlighted in red for clarity), and the sample volume (blue), so that students can more easily understand how the power M-mode and spectral displays morph in response to probe manipulation. When the student is ready to acquire an insonation (Doppler measurement at an insonation point), they are prompted to identify the artery segment. The feedback provided by the interface informs them whether they were correct and displays a 3D artery map showing the location of the sample volume and the the artery segment in which it was actually located (if not the segment targeted by the student). This feedback (Figure 3) enables students to practice in a self-directed manner without faculty oversight. The 3D map of the arteries and acoustic windows displayed during scanning (Figure 1) can be turned off by the student as their skills progress, to gradually increase difficulty and make scanning the mannequin more closely resemble patient scanning.

### Simulator Curriculum

The TCD curriculum was designed to train students in the performance of TCD ultrasound for measuring cerebral artery blood flow velocity (Table 1). In addition to didactic material on cerebrovascular circulation and the basics of TCD, students were taught how to identify and insonate the middle cerebral artery (MCA), anterior cerebral artery (ACA), ophthalmic artery (OA), basilar artery (BA), and vertebral artery (VA). Insonation of the posterior cerebral artery (PCA) was taught as optional enrichment; it was not tested. The training followed the practice standard recommended by experts in TCD and members of the American Society of Neuroimaging Practice Guidelines Committee as well as international neurosonological organizations.^1^ The curriculum emphasized virtually guided instruction in English with deliberate hands-on practice on a mannequin, leveraging established educational principles such as scaffolding and deliberate practice.^12^ The entire curriculum was delivered on the simulator. Didactic content was interspersed among practice scan cases to not only teach how to identify and insonate targeted vessels but also explain the clinical application of each procedure. Multiple-choice questions (MCQs) were included in the didactic content to vary the pace between passive and active learning and reinforce the lessons, as well as practice cases that challenge students to apply their knowledge in simulated clinical scenarios. Clinical applications of TCD including vasospasm, SCD, and optimization of signal quality during examinations were also taught.

**Table 1:** Curriculum Map: Outline of Modules and Assessment

Module/topic	Primary vessels/segments	Acoustic window(s)	Topic addressed	Use in assessment
Cerebrovascular circulation (anatomy and flow)	MCA, ACA, PCA, t-ICA, OA, VA, BA	Overview (all)	Anatomic landmarks; standard vessel coverage	MCQs only
Scanning fundamentals and signal optimization	All (per window)	All	Technique; waveform quality; depth/orientation; documentation	Implicit through hands-on tasks
Transtemporal window—MCA protocol	M1 (proximal/mid/distal as taught)	Transtemporal	Standard MCA insonation (depth, direction, labeling)	Pre-test (left MCA); posttest (bilateral)
Transtemporal window—ACA protocol	A1 (ipsilateral)	Transtemporal	Standard ACA insonation (depth, direction, labeling)	Pre-test (right ACA); posttest (bilateral)
Transtemporal window—PCA protocol	P1/P2 (as taught)	Transtemporal	PCA insonation per practice parameters	Practice only (optional enrichment)
Transorbital (transophthalmic) window—OA protocol	OA	Transorbital	OA insonation; safety considerations (power)	Posttest (bilateral)
Transforaminal window—VA protocol	VA	Transforaminal (suboccipital)	VA insonation; laterality; depth; direction	Posttest (bilateral)
Transforaminal window—BA protocol	BA proximal/mid/distal	Transforaminal (suboccipital)	BA insonation and segment labeling	Posttest (3 segments)
Knowledge checks (“Quick Questions”)	N/a	N/a	Indications; safety; documentation; protocol sequencing	Pre-test and posttest MCQs
Practice tasks and acquisition feedback loop	All per case (CTA/TCD-derived simulated cases)	All	Procedural coverage; labeling; documentation	Throughout practice; Test Mode disables guidance

Abbreviations: ACA = anterior cerebral artery; BA = basilar artery; ICA = internal carotid artery; MCA = middle cerebral artery; OA = ophthalmic artery; PCA = posterior cerebral artery; VA = vertebral artery.

Trainees followed the modules stepwise from the beginning but were encouraged to backtrack and repeat earlier ones as desired or needed to strengthen their skills, without limitation. We elected to proctor each student's first session to observe their workflow and to assist with simulator operation if needed. No oversight was provided to students in subsequent sessions.

### Assessment

Students' technical skill in performing foundational TCD was assessed using a pre-test at baseline and a posttest after completion of training. The tests were simulator-based, and they were developed with input from 3 TCD experts, 2 vascular technologists who are lead sonographers (responsible for the practical training of new TCD sonographers) at the UW and Harborview Medical Center (HMC), and the physician directing TCD education at HMC (D.S.) who was responsible for overseeing didactic and hands-on training in TCD. Based on the collective input of these experts, the tests on the simulator assessed the essential elements of a TCD examination. The experts vetted the cases chosen and the simulator interface by taking the tests themselves. The experts repeated the test after it was revised based on their feedback. The test submitted to students in this study was the revision that satisfied all 3 experts.

The MCQs on the pre-test and posttest were representative of clinically relevant portions of the curriculum, developed through an initial literature review and validated by internal undergraduate participant testing. The MCQs were designed by content experts to assess foundational knowledge constructs such as cerebrovascular anatomy and the clinical indications for TCD. While a specific score for “competence” was not defined for this foundational training, successful completion of the curriculum and substantial improvement on the posttest were considered indicators of acquired skill.

Students were asked to undergo a brief pre-test to establish their baseline skill in TCD, which consisted of 1 MCQ and insonation of the left MCA and right ACA on 1 patient case. Written instructions provided before the pre-test focused on the simulator interface: students received no instruction on the scan procedure from the proctor. In the Test Mode, cases were presented to the students without the visual guidance display. The posttest was comprehensive to assess curriculum efficacy: insonation of the left and right MCA, ACA, OA, and VA, as well as the proximal, mid, and distal segments of the BA, all on 1 patient case, and 2 MCQs based on the didactic content of the curriculum. On both the pre-test and posttest, students were allowed unlimited time to answer the MCQs and unlimited attempts to scan the targeted vessels. Each time they made an acquisition they were asked to identify the vessel they thought they insonated. The correct vessel labeling was then revealed so that testing could also be a learning experience. All attempts at insonation were recorded automatically, along with the targeted vessel, the vessel actually insonated if different, and the time stamp of the attempt.

### Data Analysis

Insonation was graded based on correct insonation of the vessel requested in the test. The principal end point was the percent of targeted vessels (2 in the pretest and 11 in the posttest) correctly insonated by each student, averaged over all students. The percent of vessels correctly insonated on the first attempt was also calculated using the time stamp recorded by the simulator on every insonation attempt (Table 2). Answers were counted as incorrect if the student failed to insonate the targeted vessel or the student did not attempt a particular vessel (Table 2). Credit was given for the correct answer on binary MCQs and for all correct answers selected on MCQs with multiple responses. Each student's MCQ score was the number of correctly selected responses expressed as a percent of the total possible responses. Results are expressed as the mean ±1 SD.

**Table 2:** Proportion of Vessels Attempted During Pretest and Posttest Assessments

Assessment	Vessel	Insonation attempted (%) (attempted, total)
Pre-test	Left MCA	54 (31, 56)
	Right ACA	7 (4, 56)
	Mean	31 (17.5, 56)
Posttest	Left MCA	93 (53, 56)
	Right MCA	79 (45, 56)
	Left ACA	82 (47, 56)
	Right ACA	65 (37, 56)
	Left OA	74 (42, 56)
	Right OA	74 (42, 56)
	BA proximal	70 (40, 56)
	BA mid	68 (39, 56)
	BA distal	51 (29, 56)
	VA left	63 (36, 56)
	VA right	65 (37, 56)
	Mean	71 (41, 56)

Abbreviations: ACA = anterior cerebral artery; BA = basilar artery; ICA = internal carotid artery; MCA = middle cerebral artery; OA = ophthalmic artery; PCA = posterior cerebral artery; VA = vertebral artery.

The mean scores on insonations and MCQs before and after course completion were compared using the paired *t* test to assess the impact of the curriculum on skill and knowledge acquisition after an assumption of normality was deemed acceptable based on visual inspection of box plots and assessment of descriptive statistics. Cohen d was calculated for the paired *t* test results for both MCQs and the TCD scans to measure the practical significance of the training effect. Comparison of medical and premedical student results was performed using the two-sample *t* test. Statistical analyses were conducted using Microsoft Excel, with significance set at *p* < 0.05.

### Standard Protocol Approvals, Registrations, and Participant Consents

The UW Human Subjects Division determined on December 21, 2023, that the proposed activity is human subjects research that qualifies for exempt status (Category 1) (IRB ID: STUDY00019390).

The study was conducted from May 24, 2024, to August 19, 2024. Recruitment was discontinued after 30 students completed the curriculum. However, students already on the waitlist were trained as time allowed. Students who completed the course were invited informally to give feedback. Students who failed to complete the course were surveyed regarding their reasons for noncompletion.

### Data Availability

Anonymized data not published within this article will be made available by request from any qualified investigator.

## Results

### Study Population

Ninety-eight students enrolled in the TCD curriculum. Of these, 3 students were rejected because of previous TCD knowledge (1 student) and because instruction on performing TCD was inadvertently provided before the pre-test (2 students). We report the data of 56 students (63% of 95% eligible) who completed the course: 12 (21%) were medical students and 44 (79%) were premedical students. No students required additional support from the educators after the initial session.

Of the 39 students who did not complete the course, the proportion who were medical students (23%) was similar to that of students who completed the course. Ten responded to our survey with the following reasons for noncompletion: health issues (10%), family obligations (10%), technical issues (10%), more enjoyable activities (10%), travel (20%), loss of interest (20%), and unexpected academic load or other commitments (40%). Each reason was selected by at least 1 student; some students gave multiple reasons.

### Technical Skill

**Figure 4:**
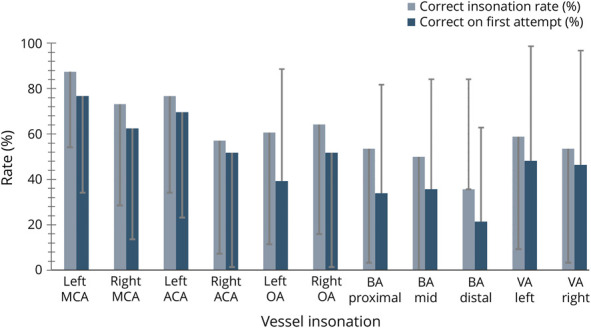
Insonation Success Rate After Training Posttest insonation rates for the middle cerebral artery (MCA), anterior cerebral artery (ACA), ophthalmic artery (OA), basilar artery (BA), and vertebral artery (VA). Error bars represent 1 SD.

**Figure 5:**
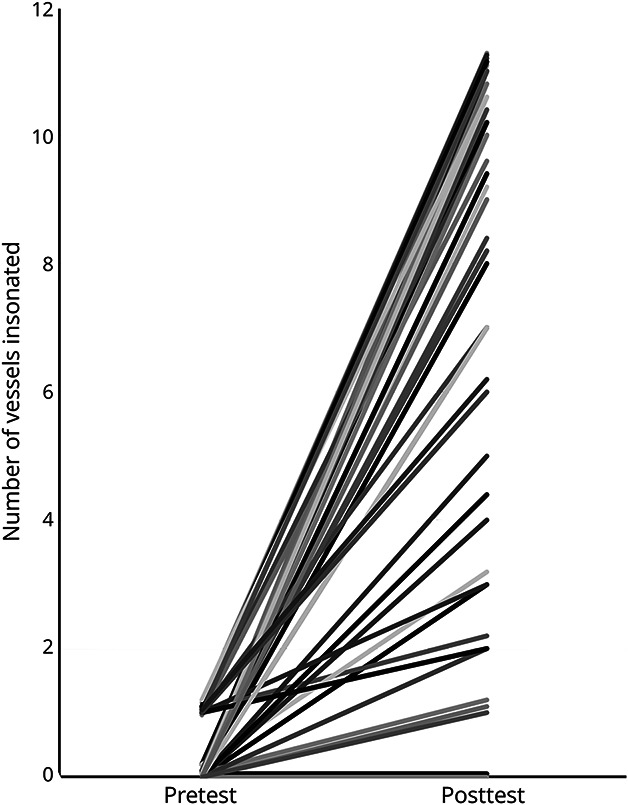
Number of Vessels Correctly Insonated Each student's data are shown at pre-test and posttest; values have been incremented slightly to aid visualization of individual students' results.

The students insonated 61 ± 34% (95% CI 52%–70%) of the targeted vessels correctly on the posttest, an improvement of 45 ± 38% from the pre-test (16 ± 24% (95% CI 9%–23%), N = 56, *p* < 0.0001, d = 1.5). The mean rate of correct insonations on students' first attempts was 49 ± 31% (95% CI 41%–57%) on the posttest, an improvement of 34 ± 38% from the pretest (15 ± 23% (95% CI 9%–21%), *p* < 0.0001, d = 0.9) (Figure 4). The proportion of students attempting to insonate the left MCA nearly doubled to 93% after training on the simulator compared with 54% before training (Table 2). The large SD was due to the range of improvement in scores. The number of vessels correctly insonated increased from pretest to posttest in all but 2 students (Figure 5). Students struggled most with the distal BA and the VA.

### Cognitive Skill (Knowledge)

For the study population as a whole, the average score on the MCQs was 64 ± 19% (95% CI 59%–69%) on the posttest, an improvement of 57 ± 30% from the pretest (7 ± 26% (95% CI 0%–14%), N = 56, *p* < 0.0001, d = 2.5).

### Impact of Education on Skill

The mean rate of correct insonations for premedical students (n = 44) was 64 ± 34% on the posttest, an improvement of 50 ± 37% from the pre-test (15 ± 23%, *p* < 0.0001, d = 1.7). For medical students (n = 12), the mean rate of correct insonations was 48 ± 33% on the posttest, an improvement of 28 ± 41% from the pre-test (21 ± 26%, *p* = 0.039, d = 0.9). The improvement among medical students (28 ± 41%) was slightly but not significantly poorer (*p* = 0.053) than among premedical students (50 ± 37%). The students did not differ significantly by education level in the insonation rate on either pre-test or posttest.

Regarding cognitive skills, the mean rate of correct MCQ answers for premedical students was 64 ± 20% on the posttest, an improvement of 57 ± 31% from the pre-test (7 ± 26%, *p* < 0.0001, d = 2.5). For medical students, the mean rate of correct MCQ answers was 68 ± 15% on the posttest, an improvement of 59 ± 27% from the pre-test (8 ± 29%, *p* < 0.0001, d = 2.6). There was no significant difference in posttest insonation skill or knowledge between the 2 groups.

### Course Duration

The course including pre-tests and posttests took an average of 2.6 ± 1.0 hours to complete. Medical students completed the course in just 1.9 ± 0.7 hours, nearly an hour faster (*p* < 0.02) than premedical students, who took 2.8 ± 1.0 hours.

### Feedback

Nine students emailed feedback and commented on the difficulty of learning the anatomy, and 6 found typographic errors or software bugs. Two suggested changes to the user interface: 1 reported being surprised that the vessels were concealed on the posttest. We have now edited the user interface to notify trainees that visual guidance is for training only and to urge them to practice with the vessels concealed. The other reported that the PCA was missing. Because the PCA was present in the assigned case, the student's report indicates that they were practicing on other cases—an indication that the curriculum was encouraging. This qualitative feedback, though limited, provides valuable user experience insights for future curriculum refinement. For example, the comment on anatomic difficulty suggests that additional preparatory materials could be beneficial.

## Discussion

Transcranial Doppler implementation into clinical practice has been significantly limited. The American Heart Association and the American Stroke Association and other professional societies recommend TCD monitoring for vasospasm in patients with aneurysmal subarachnoid hemorrhage (aSAH).^3^ However, data from 1,000 hospitals in the Nationwide Inpatient Sample showed that only 1.5% of aSAH discharges involved TCD monitoring from 2002 to 2011, reflecting low clinical implementation.^8^ Similarly, despite recommendations by the American Society of Hematology,^14^ the American Association of Family Practice,^15^ and the National Heart, Lung, and Blood Institute,^16^ TCD screening of children with SCD has been suboptimal, despite trial-proven benefits in reducing stroke.^17-19^ A database and chart review in Georgia revealed that only 47% of 125 children with SCD underwent TCD screening.^20^ Data of 3,352 patients in a nationwide Medicaid database revealed that only 47% and 38% of children and adolescents aged 2–9 and 10–16 years, respectively, received TCD screening in 2019.^21^ Despite the well-established applications of TCD in perioperative neuroscience^22^ and evidence for its utility even in patients with coronavirus infection,^23^ the clinical utilization of TCD remains low.^24^

Underutilization of TCD is likely due to a lack of adequately trained practitioners. Unfortunately, TCD is more challenging than other ultrasound applications and requires considerably more practice.^25^ TCD has been described as “the most complex physiologic test in vascular medicine,” with “few people mastering the technique over the past quarter of a century.”^4^ The standard peer-to-peer training has proven inadequate to produce enough TCD practitioners with the requisite skill level.^4,26^ One study reported that inconsistent training and varying experience levels among operators has led to unreliable TCD assessments, particularly in critical care settings where accurate readings are essential.^6^ The DISPLACE study^26^ reported poor TCD performance quality across 26 centers: in 52% of reports, it was impossible to identify which velocity measurement was used to define abnormal results.

The results of this study demonstrate the efficacy of our simulator-based curriculum in teaching medical and premedical students without experience to insonate intracerebral arteries. Although the posttest scores of 61% for correct insonation and 64% for the knowledge test do not represent mastery or even competence, they demonstrate a remarkable improvement from the pre-test baseline in most students (Figure 5). It is also important to consider that this was a voluntary, ungraded course without extrinsic incentives for achieving a perfect score. The curriculum's success should be viewed as providing a strong and rapidly acquired foundation on which clinical competence can be built, with the expectation that scores would improve further in a formal certification or graded context. Indeed, simulation is the only means of assessing whether a student correctly insonates a targeted intracerebral artery using TCD. The simulator was also effective at imparting knowledge, as evidenced by the improved scores on the MCQs.

An unanticipated benefit was our curriculum's efficiency: a significant gain in skill and knowledge was achieved in just 2.6 ± 1.0 hours on average (including test time). Based on these results, expansion of the curriculum content and practice cases is warranted to enable trainees to reach higher levels of skill and to introduce them to pathology. The simulator cannot completely train to competence because clinical competence requires clinical training from scanning patients. However, the simulator's assessment metrics may help faculty identify trainees who have achieved sufficient technical skill to progress to and benefit from clinical training, flag trainees who need additional instruction, or help grade the efficacy of a curriculum or program. For example, the faster completion time by medical students suggests the need to expand instruction in anatomic concepts and medical terminology when training premedical students. Our group is examining the efficacy of simulator-based training for physicians practicing clinical neuroscience (neurology, neurosurgery, neuroanesthesiology, neurocritical care, etc), and faculty at the UW School of Medicine are interested in using TCD simulation to help students learn neuroanatomy.

Our findings are consistent with previous studies that demonstrate the efficacy of simulation-based training in improving technical and cognitive skills in medical ultrasound.^10,11,27-30^ For example, technical skills and knowledge in focused cardiac ultrasound improved significantly through a simulator-based curriculum that provided immediate feedback.^27^ Integrating the educational techniques of scaffolding and deliberate practice into ultrasound training enhanced student learning.^11^ A meta-analysis reported the superiority of simulator-based medical education with deliberate practice to traditional clinical training.^31^ A review of simulation recommends its use in neurocritical care training to enhance competency in acquisition and interpretation through practicing emergency scenarios.^28,32^ These studies, along with ours, underscore the value of simulation-based education in providing a controlled, reproducible environment for skill acquisition.

Standard TCD training involves didactic lectures, followed by supervised hands-on practice on patients, a time and resource-intensive process. Certification of vascular technologists by the Intersocietal Accreditation Council requires experience with 100 TCD cases.^33^ Certification of physicians to interpret TCDs requires them to interpret 100 TCD cases under supervision or undergo a 40-hour CME training with at least 8 hours of supervised clinical experience observing or participating in an accredited vascular laboratory.^33^ The present results indicate that the simulator can serve as a valuable adjunct to TCD training. Our curriculum differs by providing a standardized, self-directed learning environment that allows for unlimited deliberate practice without faculty supervision or risk to patients. While it borrows elements from standard training, such as adherence to established scanning protocols, its primary advantage is scalability and accessibility, directly addressing the training bottleneck that limits TCD availability.

We acknowledge that comprehensive TCD performance involves advanced clinical interpretation beyond the scope of this introductory module. However, vessel insonation is the critical first step and a significant barrier for novices. We, therefore, designed a curriculum focused on establishing this foundational skill, which is prerequisite for any subsequent clinical application such as assessment of stroke risk in children with SCD.^19^ Future work will focus on expanding the curriculum to transition learners from this procedural task training to clinical practice. This expansion can be easily accomplished by adding the requisite didactic materials. Indeed, our simulator is already equipped to develop a vasospasm curriculum: it has a library of 24 vasospasm cases with diverse pathology and tools for calculation of Lindegaard and Sviri ratios (Appendix). This planned future module will guide trainees through the process of examining a simulated patient, interpreting the findings, and making a diagnosis. Furthermore, the curriculum can be adjusted for advanced learners by including cases of anatomic variants such as fetal origin PCA and by engineering variability in the size and shape of the temporal acoustic window. This tiered approach will allow learners to acquire requisite technical skills first and then apply them in a risk-free, simulated clinical environment. The intent guiding the design of the TCD simulator was applicability to the full spectrum of training from students to practicing physicians across various specialty areas. The simulator's skill metrics enable standardized testing to assess trainee progress toward certification.

Forty-one percent of the students who began the course did not return to complete it. Although disappointing, it is understandable because these students volunteered for medical training during their summer vacation, and the course is an optional enhancement rather than a required part of their academic or career development. Among the students who did not complete the course, reasons for noncompletion were varied and external. One student cited technical issues but also had unexpected academic loads or other commitments, indicating that the training itself was not the primary barrier.

Many students successfully insonated the MCA on the pre-test, but they rarely attempted the right ACA, and none succeeded. This is probably because the simulator's instructions were illustrated with an insonation of the MCA but not the ACA. We surmise that the pre-test success rate for insonating the MCA is artificially elevated, and that text-only instructions would have resulted in a success rate closer to that of the ACA (0%). This lack of clear prebriefing on all expected vessels in the pre-test is a limitation that may confound the pre-test results.

In the interest of minimizing course duration, the posttest only presented 1 case, one that had not appeared before. Increasing the number and variety of cases would provide more generalizable results.

These results pertain to intermediate skill acquisition. Future studies with delayed follow-up and testing are needed to assess long-term skill retention.

Another potential limitation is that skill acquisition was not confirmed on live human patients, and our testing protocol provided immediate feedback, allowing learners to make repeated attempts until successful. However, it is crucial to note that the simulator is built on real patient CTA and TCD scans, not theoretical models. This provides a high-fidelity environment that closely mimics human anatomy and hemodynamics, making the skills acquired directly translatable. The rationale for providing immediate feedback during testing was to reinforce the learning process. To distinguish between direct skill acquisition from the curriculum and learning through trial-and-error methods, we report 2 metrics: first-attempt accuracy and overall accuracy. First-attempt accuracy serves as a direct measure of the skill without feedback on a given scan. The improvement in overall accuracy, by contrast, demonstrates the efficacy of the simulator's interactive feedback in enabling rapid, self-directed skill refinement. Although this approach limits the interpretability of the overall success rate as a standalone measure of final competence, it highlights the dual strengths of the curriculum in both teaching the procedure and facilitating mastery through iterative practice.

The results of this study demonstrate that our simulation-based curriculum is an effective tool for teaching novice students the foundational skills of TCD. The students' success in vessel insonation underscores the simulator's potential to help address the current gap in TCD training by providing a highly effective foundational solution that enables self-directed practice, provides immediate feedback, avoids disturbing sick patients, and minimizes the need for faculty oversight. This expansion is vital, given TCD's unique role in neuromonitoring, and its widespread adoption has the potential to improve neurologic outcomes by ensuring more accurate and timely assessments of cerebral hemodynamics. Moving forward, integrating this curriculum into standard formal training programs could significantly enhance the quality and availability of TCD diagnostics in clinical practice, ultimately leading to better patient care and outcomes, particularly in regions where access to expert instruction is limited. Although this curriculum is not equivalent to the formal training required for Registered Physician in Neurovascular Interpretation (RPNI) neurosonologist certification, it represents a crucial first step. Next steps include exploring embedded learning within residency programs and assessing skill transfer through practice on standardized patients.

## Glossary

ACA: anterior cerebral artery
aSAH: aneurysmal subarachnoid hemorrhage
BA: basilar artery
CFD: computational fluid dynamics
CTA: CT angiography
HMC: Harborview Medical Center
MCA: middle cerebral artery
MCQ: multiple-choice question
OA: ophthalmic artery
PCA: posterior cerebral artery
SCD: sickle cell disease
TCD: transcranial Doppler
UW: University of Washington
VA: vertebral artery
